# Targeting Pin1 for Modulation of Cell Motility and Cancer Therapy

**DOI:** 10.3390/biomedicines9040359

**Published:** 2021-03-31

**Authors:** Hsiang-Hao Chuang, Yen-Yi Zhen, Yu-Chen Tsai, Cheng-Hao Chuang, Ming-Shyan Huang, Michael Hsiao, Chih-Jen Yang

**Affiliations:** 1 Division of Pulmonary and Critical Care Medicine, Department of Internal Medicine, Kaohsiung Medical University Hospital, Kaohsiung Medical University, Kaohsiung 80708, Taiwan; hsianghao.chuang@gmail.com (H.-H.C.); 1010362@kmuh.org.tw (Y.-C.T.); 1040239@kmuh.org.tw (C.-H.C.); 2 Division of Nephrology, Department of Internal Medicine, Kaohsiung Medical University Hospital, Kaohsiung Medical University, Kaohsiung 80708, Taiwan; a0928306234@gmail.com; 3 Department of Internal Medicine, E-Da Cancer Hospital, School of Medicine, I-Shou University, Kaohsiung 82445, Taiwan; Ed110209@edah.org.tw; 4 Genomics Research Center, Academia Sinica, Taipei 11529, Taiwan; 5 Department of Respiratory Therapy, College of Medicine, Kaohsiung Medical University, Kaohsiung 80708, Taiwan; 6 School of Medicine, College of Medicine, Kaohsiung Medical University, Kaohsiung 80708, Taiwan

**Keywords:** Pin1, *cis*-*trans* isomerization, tumorigenesis, cell motility, metastasis, cancer therapeutics

## Abstract

Peptidyl-prolyl *cis-trans* isomerase NIMA-interacting 1 (Pin1) specifically binds and isomerizes the phosphorylated serine/threonine-proline (pSer/Thr-Pro) motif, which leads to changes in protein conformation and function. Pin1 is widely overexpressed in cancers and plays an important role in tumorigenesis. Mounting evidence has revealed that targeting Pin1 is a potential therapeutic approach for various cancers by inhibiting cell proliferation, reducing metastasis, and maintaining genome stability. In this review, we summarize the underlying mechanisms of Pin1-mediated upregulation of oncogenes and downregulation of tumor suppressors in cancer development. Furthermore, we also discuss the multiple roles of Pin1 in cancer hallmarks and examine Pin1 as a desirable pharmaceutical target for cancer therapy. We also summarize the recent progress of Pin1-targeted small-molecule compounds for anticancer activity.

## 1. Introduction

Cells respond to extracellular and intracellular stimuli and elicit various regulatory mechanisms such as epigenetic regulations, allosteric regulations and post-translational modifications (PTMs) to control signal transduction [1,2,3]. PTMs are critical and complex regulators, which control many cellular processes by changing protein structure, thereby causing alteration in enzyme activity, protein interaction, protein compartmentalization, protein processing, and protein stability. Protein phosphorylation is the prevalent modification which regulates biologic processes in eukaryotic cells [4]. Phosphorylation of serine or threonine residues preceding a proline (pSer/Thr-Pro) is one of the most frequent PTMs and it occurs in a wide range of proteins which are usually involved in cell cycle progression in eukaryotic cells [5,6]. This modification is catalyzed by proline-directed kinases, which include cyclin-dependent protein kinases (CDKs), mitogen-activated protein kinases (MAPKs) such as extracellular signal-regulated kinases (ERKs)/c-Jun N-terminal kinases (JNKs)/p38 mitogen-activated protein kinases (p38 MAPKs), glycogen synthase kinase-3 (GSK3) and polo-like kinases (PLKs) [7,8].

In addition to phosphorylation, the *cis/trans* isomerization of prolyl peptide bonds is an aspect of conformational changes. Proline is a unique residue that can adopt a *cis* or *trans* configuration; the configuration switch of its peptide bonds is tightly controlled by prolyl *cis-trans* isomerization and plays a critical role in multiple cellular processes [8]. These prolyl *cis-trans* isomerizations are catalyzed by peptidyl-prolyl *cis/trans* isomerases (PPIases). There are four PPIase subfamilies: cyclophilins, FK506-binding proteins (FKBPs), parvulins, and protein phosphatase 2A phosphatase activators (PTPAs) [9,10]. Peptidyl-prolyl *cis-trans* isomerase NIMA-interacting 1 (Pin1), a member of the parvulin subfamily, is a unique enzyme that specifically binds and isomerizes phosphorylated serine-proline or phosphorylated threonine-proline (pSer/Thr-Pro) motifs, thereby controlling diverse biological processes [8,10,11,12]. Pin1-mediated phosphorylation dependent protein isomerization is a novel post-phosphorylation regulatory mechanism [13,14,15].

The Pin1 gene is localized on chromosome 19p13.2 and encodes the Pin1 isomerase, which consists of 163 amino acids of approximately 18 kDa [13,15]. Pin1 consists of an N-terminal protein-binding WW domain and a C-terminal PPIase domain. The WW domain preferentially binds to pSer/Thr-Pro containing peptide sequences, while the catalytic domain isomerizes the prolyl bond in the pSer/Thr-Pro motif [14,16]. Pin1 was initially identified as being involved in cell cycle progression, but mounting evidence has shown that Pin1-mediated prolyl isomerization plays a crucial role in diverse biological processes, including cell cycle regulation, cell growth, differentiation, immune responses, stemness, and even tumorigenesis [8,17,18,19]. Moreover, Pin1 is commonly overexpressed in most cancers and high levels of Pin1 expression is correlated with poor prognosis in various cancers [10,11,20,21,22,23,24]. An increasing body of evidence has shown that Pin1 promotes tumor initiation, development, and resistance to cell death as well as enabling replicative immortality by upregulating oncogenes and proliferating-promoting factors or downregulating tumor suppressors and proliferation-suppressing factors [10,11,12,19]. Therefore, Pin1 is regarded as an intriguing target for cancer therapy.

The current study sets out to discuss the critical role of Pin1 in cancer, and summarizes the recent findings, which show that Pin1 plays multiple roles in tumorigenesis. At present, Pin1 is a desirable pharmaceutical target for cancer therapy, and the present study summarizes the recent progress of Pin1-targeted small-molecule compounds for cancer therapy.

## 2. Dysregulation of Pin1 in Cancer

### 2.1. Regulation of Pin1 Expression at the Transcriptional, Post-Transcriptional and Post-Translational Levels

A growing amount of evidence has shown that Pin1 is highly expressed in most cancers and is correlated with poor prognosis in human cancers. The expression of Pin1 is controlled by multiple regulatory mechanisms. Initially, Pin1 expression is transcriptionally regulated by CDK-retinoblastoma protein (Rb)-E2F axis [25,26,27]. A mechanistic investigation revealed that Pin1 transcription is activated by the E2F family, which bind to the E2F binding sites of the PIN1 promoter [25]. It also revealed that activated HER2 and Ras signaling can stimulate Pin1 expression in breast cancer development. However, hypo-phosphorylated Rb binds to the E2F transcription factor and coincides with transcriptional inactivation of Pin1 expression [26,28], while CDK-mediated phosphorylation of Rb would trigger the dissociation from the Rb-E2F complex, leading to increased E2F transcriptional activity and Pin1 expression. Gubern et al. found that p38 MAPK-mediated Rb phosphorylation results in Rb insensitive to CDK inactivation and increases Rb-E2F affinity to repress E2F-activated transcription [29]. Furthermore, E2F-induced Pin1 transcription is also activated by other transcriptional factors, such as C/EBPα-p30, a mutant of transcription factor C/EBPα, which induces Pin1 expression by recruiting E2F1 to the PIN1 promoter [30]. In addition to E2F-mediated Pin1 expression, Notch1 binds to the PIN1 promoter and triggers Pin1 transcription [31]. Conversely, tumor suppressors such as p53, AP4, and BRCA1 act as transcription repressors which inhibit Pin1 expression [32,33,34].

In addition to transcriptional regulation, Pin1 expression is also regulated at the post-transcriptional level, which primarily affects mRNA stability and protein translation. mRNA stability is controlled by microRNAs (miRNAs), a class of small non-coding RNAs that reduce gene expression by suppressing translation or destabilizing target RNAs [35,36]. Some miRNAs have been identified to reduce Pin1 expression and suppress tumorigenesis, including miR-140-5p, miR200b, miR200c, miR296-5p, miR370, miR-628-5p, and miR874-3p [37,38,39,40,41,42,43]. Interestingly, the total miRNA expression profile is generally downregulated in tumors and this could lead to Pin1 upregulation in cancers [44,45]. Furthermore, Pin1 might reduce miRNA biogenesis by modulating the function of exporin-5 (XPO5) in cancers [46].

Post-translational regulation, which alters protein stability, is another way to control Pin1 expression level. PLK1 phosphorylates Pin1 at the Ser65 residue in the PPIase domain and prevents Pin1 ubiquitination, which increases its protein stability in human cancer cells [47]. SENP1, a SUMO protease, removes SUMOylation from Pin1 and increases its stability [48]. Pin1 has been previously identified as being modified by acetylation in a large-scale proteomics screening [49]. Furthermore, GRK2-HDAC6-mediated deacetylation of the lysine 46 residue on Pin1 enhances Pin1 stability and promotes higher substrate affinity and isomerase activity in breast cancer cells [50]. In summary, Pin1 is prevalently upregulated in many human cancers through transcriptional, post-transcriptional, and post-translational regulations.

### 2.2. Regulation of Pin1 Function

In addition to the expression level, post-translational regulation is a crucial way of controlling Pin1 function. To date, post-translational modifications of Pin1 have been identified as phosphorylation, SUMOylation, oxidation, acetylation and ubiquitination [3]. These modifications change the Pin1 structure at the corresponding sites, leading to alterations in protein stability, cellular localization, protein interactions and enzymatic activity under both physiological and pathological conditions. Some of the modifications mentioned above affect the protein stability of Pin1. cAMP-dependent protein kinase A (PKA) phosphorylates Pin1 at Ser16 and disrupts its substrate binding activity [51]. Ser16 is a critical amino acid residue in the WW domain, which is responsible for the binding of phosphorylated substrates, so this conformation change may influence the binding ability of Pin1 [51]. Aurora A also phosphorylates Pin1 at Ser16 and abolishes its binding ability to Bora, leading to increased Bora protein stability and perturbation of mitotic progression [52]. However, ribosomal protein S6 kinase 2 (RSK2) forms a complex with Pin1 and phosphorylates its Ser16, leading to increased 12-O-tetradecanoylphorbol-13-acetate (TPA)-induced cell transformation in JB6 CI41 cells [53]. Furthermore, MAP3K-related serine/threonine kinase COT has been reported to phosphorylate Pin1 at Ser16, which consequently stabilizes cyclin D1 protein and promotes tumorigenesis in breast cancer cells [54]. Pin1 plays a crucial role in the modulation of the cell cycle [55] and it is tightly regulated during the cell cycle to control for the presence of physiological or pathological conditions. While the same modification may occur on the same residue, the different cellular context or the different binding partner can drive distinct biological processes. In addition, MLK3, a member of the MAP3K family, was found to phosphorylate Pin1 at Ser138 which promotes its catalytic function, nuclear translocation, and increased protein stability [56]. Therefore, the MLK3-Pin1 pSer138 axis promotes tumorigenesis in breast cancer through enhancement of cell cycle progression and centrosome amplification. Conversely, DAPK1, a tumor suppressor, phosphorylates Pin1 at Ser7, which leads to inhibition of catalytic activity and blockage of nuclear localization [57,58,59]. In addition to phosphorylation, SUMOylation at Lys6 and Lys 63 of Pin1 inhibits its binding ability and catalytic activity [48]. In addition to protein stability, SENP1, a SUMO protease, deSUMOylates Pin1 leading to increased Pin1 activity and promoted centrosome overduplication and cell transformation during tumorigenesis [48]. As mentioned above, HDAC6-mediated de-acetylation of the lysine 46 residue on Pin1 not only promotes Pin1 stability but also enhances its substrate binding activity and enzymatic activity [50]. Taken together, these conformational modifications, which are induced by tumor-promoting signals or tumor-suppressive signals, regulate Pin1 functions to control diverse biological processes.

## 3. Dysregulation of Pin1 Signaling in Tumorigenesis

**Figure 1:**
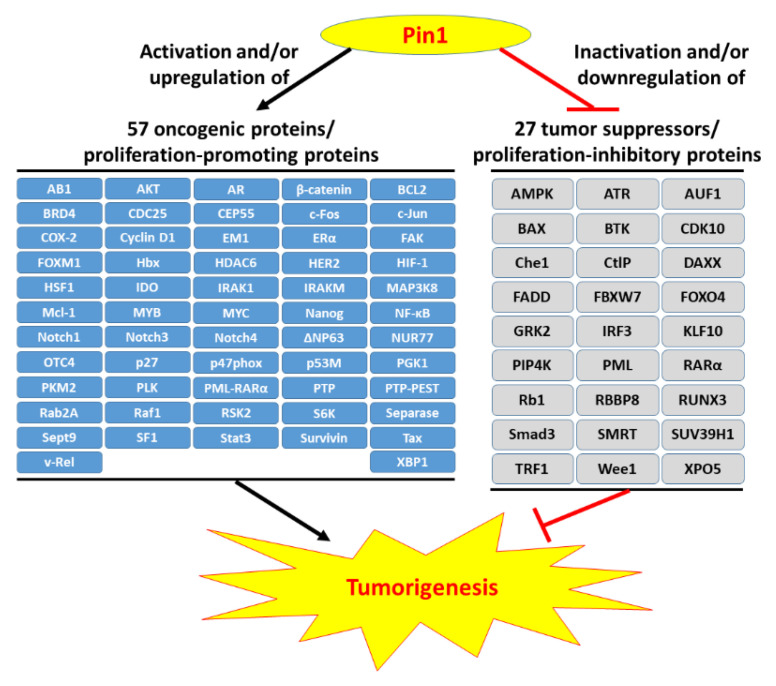
The targets of peptidyl-prolyl cis-trans isomerase NIMA-interacting 1 (Pin1) involved in cell propagation and tumorigenesis. Pin1 promotes tumorigenesis via upregulation of >50 oncogenes or proliferation-promoting proteins and downregulation of >20 tumor suppressors or proliferation-inhibitory proteins.

**Figure 2:**
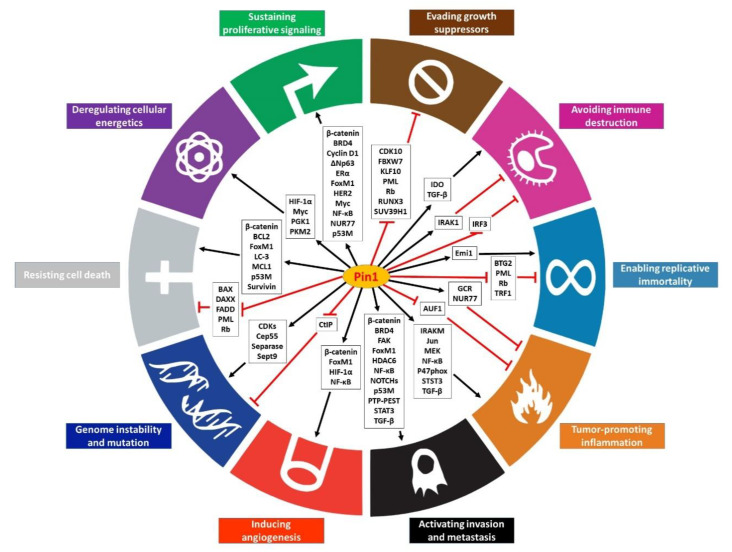
Pin1 acts as the signaling hub in cancer hallmarks. A summary of how Pin1 could contribute to tumorigenesis is illustrated in this figure where Hanahan and Weinberg proposed the hallmarks of cancer which contain ten major biological capabilities [61]. Pin1 contributes to all of these aberrant properties by exaggerating multiple cancer-driving pathways and regulating the function of the downstream substrates.

Pin1 is widely overexpressed in human cancers and its reduction significantly represses cancer progression. Pin1 is associated with the development of various cancers and is therefore regarded as a molecular timer of the cell cycle [55]. High levels of Pin1 are correlated with a higher proliferative capacity. Current studies have shown that Pin1 upregulates dozens of proliferation-promoting factors and inhibits dozens of proliferation-suppressing factors (Figure 1) [10,11,12]. However, Pin1 promotes tumorigenesis not only through enhancement of the cell’s proliferating ability but also via other tumor-promoting processes. Hanahan and Weinberg elaborately summarized the breakthrough in our understanding of the hallmarks of cancer, which are composed of ten major cancer capabilities [60,61]. The ten cancer capabilities include sustaining proliferative signaling, evading growth suppressors, resisting cell death, enabling replicative immortality, inducing angiogenesis, activating invasion and metastasis, genome instability and mutation, tumor-promoting inflammation, reprogramming energy metabolism, and evading immune destruction [61]. An increasing body of evidence has indicated that Pin1 acts as a central hub in the promotion of tumorigenesis by augmenting these cancer capacities (Figure 2). In this section, we summarize the regulatory mechanisms of Pin1 that contribute to these cancer capabilities.

### 3.1. Pin1 Enhances Proliferative Signaling

Cell cycle progression is tightly controlled in normal cells which are under various intracellular and extracellular signals stimuli. However, cancer cells exhibit dysregulation of cell cycle progression and sustain proliferative activity [62]. Pin1 was originally identified as a regulator of mitosis [13] and many previous studies have indicated that it drives multiple proliferation-promoting pathways in cancer development [55]. From observations of Pin1 knockout mice, Pin1 was identified as controlling cyclin D1 stability and driving cell proliferation [17,24]. CDKs are known to be involved in the control of cell cycle progression [63,64], therefore Pin1 and cyclin D1 form a regulatory positive feedback loop. Wee1 and Myt1 have been revealed to phosphorylate CDK1 and CDK2 at Thr14 and Tyr15, respectively, which leads to inhibition of CDKs activity and prevents cell cycle progression. However, Pin1 can interact with Myt1 and Wee1, and regulate their activity to modulate cell cycle progression [65].

β-catenin is known to promote cell proliferation in normal cells and cancer cells [66,67]. Pin1 has been found to control the stability of and subcellular localization of β-catenin by inhibiting its interaction with adenomatous polyposis coli protein (APC) [68]. Therefore, upregulation of Pin1 promotes prostate cancer cell proliferation and migration through activation of Wnt/β-catenin signaling [69]. In breast cancer, high expression of estrogen receptor α (ERα) is correlated with a poor prognosis due to abnormal cellular proliferation. Mechanistic investigation revealed that ERα promotes cell proliferation through suppression of p53/p21 and upregulation of proliferating cell nuclear antigen (PCNA) and proliferation-related Ki-67 antigen (Ki-67) in MCF-7 cells [70]. In breast cancer, Pin1 could promote cell proliferation through increased protein stability [71], DNA binding affinity [72] and transcriptional activity of ERα [73]. Interestingly, there is typically high expression of both Pin1 and HER2 in most breast cancers. Not only does HER2 promote Pin1 expression [25], but Pin1 also upregulates HER2 expression by inhibiting its ubiquitination [74] and destabilizing its transcriptional corepressor SMRT [75].

Myc controls the expression of CDKs, cyclins and E2F transcription factors to govern cell cycle progression, especially in tumor development [76,77]. Pin1 recruits Ser62-phosphorylated Myc to its co-activators and protects its stability, thereby enhancing Myc transcriptional activity. The Myc-activated signaling cascade is involved in cell growth and metabolism and can enhance the proliferative activity in tumor cells [78]. Nuclear factor (NF)-κB signaling also promotes cancer cell proliferation [79]. Pin1 is known to activate the NF-κb signaling pathway by promoting nuclear accumulation of p65 and protecting p65 from E3 ligase SOCS-1-mediated ubiquitination and degradation [80]. Pin1-promotion of proliferation via the NF-κB signaling pathway has been found in various cancers [81,82,83,84]. Additionally, ΔNp63, a potent oncogene, stimulates the expression of the Wnt receptor Fzd7, thereby enhancing Wnt signaling and maintaining dysregulated cell proliferation for tumorigenesis [85,86]. Pin1 upregulates ΔNp63 expression, which enhances cancer cells proliferation by blocking inhibition of E3 ligase WWP1-mediated ubiquitination and degradation [87]. Moreover, Pin1 also upregulates many proliferation-inducing factors, such as bromodomain-containing protein 4 (BRD4), c-Jun, FoxM1, NUR77, and XBP1 [88,89,90,91,92].

In addition to tumor cells, Pin1 also promotes the proliferation of nontumorous cells [17,93,94,95]. These results suggest that Pin1 is a potential target for the treatment of neoplastic diseases.

### 3.2. Pin1 Downregulates Dozens of Tumor Suppressors

The cell cycle is tightly controlled to maintain the genome stability under normal conditions. There are negative regulatory mechanisms which control cell proliferation. Tumor suppressor genes play critical roles in these mechanisms to prevent dysregulated proliferation and neoplasia. However, cancer cells adopt various mechanisms to circumvent these blockades and checks.

The two prototypical tumor suppressors, p53 protein and Rb protein, act as the central nodes which cooperate with the complementary cellular program to control cell fate towards proliferation, quiescence or apoptotic processes [61]. Many studies have indicated that Pin1 promotes p53 stability and functions after genotoxic insults [96,97,98,99]. Pin1 is even regarded as a tumor suppressor [55]. However, p53 is often mutated (p53M) in cancer cells. Growing evidence has shown that Pin1 promotes the p53M-induced gain of new functions (GOFs), leading to aggressive cancers [100,101,102]. It has also been indicated that TP53 mutations are usually correlated with poor prognosis in human cancers [100]. Rb directly binds and inhibits the E2F transcription factor to arrest the cell cycle upon DNA damage [103]. However, Rb is usually inactivated through downregulation and/or hyperphosphorylation by CDKs which are activated by increased cyclin D1 in cancer cells [24,63,64].

Additionally, promyelocytic leukemia protein (PML) is a well-known tumor suppressor that is involved in various cellular processes including apoptosis, viral infection, senescence, DNA damage repair, and cell cycle regulation but is frequently mutated in cancers [104]. Pin1 can destabilize PML to promote the proliferation of breast cancer cells [105]. Pin1 promotes E3 ligase KLHL20-mediated degradation of PML to enhance prostate cancer progression [106]. Pin1 also stabilizes the oncogenic fusion protein PML-RARα [107], which disrupts the localization of wild-type PML from nuclear bodies to numerous micro speckles [108] and induces a maturation arrest in promyelocytic leukemia [109]. Pin1 inhibition by small molecular compounds suppresses cancer cell proliferation and increases the protein levels of PML and SMRT [110]. Additionally, Pin1 downregulates numerous tumor suppressor genes such as CDK10, E3 ligase FBXW7, Kruppel-like factor 10 (KLF10), runt-related transcription factor 3 (RUNX3), and suppressor of variegation 3–9 homolog 1 (SUV39H1) to drive multiple oncogenic signaling pathways [111,112,113,114,115].

### 3.3. Pin1 Helps Cancer Cells Resist Cell Death Signaling

Neoplastic cells with dysregulated proliferation activity harbor high levels of DNA damage due to replication stress. Apoptosis is the primary method of programmed cell death, which serves as an elaborate blockade to cancer development and is a program designed to eliminate genetically damaged cells [116,117]. However, cancer cells are able to inhibit pro-apoptotic signals and activate anti-apoptotic signals, which allow them to survive under various cytotoxic stress. Pin1 is a critical defense against genotoxic insult-induced apoptosis for cancer cells [118].

For one thing, Pin1 can suppress various pro-apoptotic signals. Following apoptotic stimulation, BAX and BAK-induced programmed cell death occurs via permeabilization of the outer mitochondrial membrane (OMM) and the subsequent release of cytochrome c [119]. Following cytokine stimulation, Pin1 suppresses the mitochondrial translocation of BAX to inhibit apoptosis in human eosinophils [120]. Furthermore, the death-associated proteins DAXX and FADD are two critical molecules which mediate the extrinsic cell death pathway [121,122]. Pin1 inhibits DAXX-induced apoptosis by downregulation of DAXX through the ubiquitin-proteasome pathway in glioblastoma cells. [123]. In addition, Pin1 disrupts Fas-FADD-mediated apoptosis through isomerization-facilitated dephosphorylation of FADD Ser194, leading to sequestration of FADD into the cytoplasmic location of eosinophils [124]. DNA damage-induced apoptosis is also mediated by other surveillance proteins, such as p53, PML, and Rb. However as mentioned above, PML and Rb are downregulated by Pin1 in various human cancers. Pin1 strengthens p53-induced apoptosis [97,98] but promotes the p53M-induced tumorigenic functions [100,101,102].

Pin1 can also activate the anti-apoptosis signals. The B-cell lymphoma 2 (BCL-2) family of proteins controls cell fate between either survival or commitment to mitochondria-mediated programmed death. The pro-survival BCL-2 family proteins such as BCL-2, BCL-XL and myeloid cell leukemia-1 (Mcl-1) reduce BAX and BAK-mediated cell death by controlling the retro-translocation of BAX and BAK from the mitochondria into the cytosol [125,126]. Evidence has shown that Pin1 mediates dephosphorylation of BCL-2 to enhance its stabilization and anti-apoptosis activity [127]. Interestingly, reinforcement of BCL-2 and Myc signaling would strengthen apoptosis abrogation, as well as sustaining proliferation for malignancy [128]. Furthermore, Pin1 stabilizes Mcl-1, which promotes chemoresistance, thereby making it positively correlated with poor survival in human breast cancer [129]. Pin1 also contributes to cisplatin resistance through upregulation of the FoxM1 and Wnt/β-catenin signaling pathway in cervical cancer cells [130]. In addition, Pin1 enhances tamoxifen resistance through upregulation of LC-3, leading to the induction of protective autophagy in breast cancer cells [131]. Pin1 also inhibits apoptosis by mediating the anti-apoptotic function of survivin, which blocks pro-caspase-9 function in hepatocellular carcinoma [132].

### 3.4. Pin1 Provides the Cancer with Replicative Immortality

Most normal cells are able to pass through only a limited cycles of successive cell divisions and then enter the status of replicative senescence. After senescence bypass, cells undergo crisis, leading to cell death by mitotic telomere deprotection [133]. These barriers are adopted to block tumor formation, whereas the cells possess unlimited replicative activity by sustaining telomeres protecting the ends of chromosomes and this program is termed immortalization.

In most cancer cells, telomerase is reactivated to maintain the extending telomeric DNA while telomeric repeat-binding factors (TRFs) limit its elongation by binding telomere in *cis* [134]. Evidence shows that Pin1 could sustain the extending telomeric DNA through destabilizing TRF1 in cancer cells [135]. Furthermore, Pin1 also inhibits Rb- and PML-induced senescence in cancer cells [105,136]. In normal cells, the p53-responsive gene BTG2 induced replicative senescence in p53 in an independent manner followed by relocalization of Pin1 thereby inhibiting its activity [137]. Ectopic expression of Pin1 suppresses BTG2-induced senescence and this indicates that the BTG2-induced replicative senescence is through the antagonizing of the Pin1 function in normal cells. Additionally, the early mitotic inhibitor 1 (Emi1) promotes S phase entry and suppresses DNA damage-induced senescence by inhibiting APC^Cdh1^ complex [138,139]. Pin1 was known to enhance the proliferation and suppresses the senescence by stabilizing Emi1 in cancer cells [140]. BCL-2 cooperates with c-Myc to immortalize pre-B cells [141] and this cooperation would promote malignancy from abrogation of apoptosis as well as sustained proliferation in cancer cells [128].

### 3.5. Pin1 Induces Tumor-Associated Angiogenesis

Angiogenesis is tightly controlled under physiological conditions. However, neoplasia or cancer cells require supplementation with a myriad of nutrients and oxygen, as well as the evacuation of metabolic waste products and carbon dioxide. Therefore, the tumor-associated neovascularization, a type of angiogenesis, occurs to satisfy these needs and help maintain blooming neoplastic development. Rapidly expanding cancer can induce continuous angiogenesis to maintain the supply of nutrients and oxygen, as well as to eliminate metabolic waste and carbon dioxide.

Mounting evidence indicates that Pin1 plays a critical role in tumor-associated angiogenesis [12,142]. Pin1 is known to promote angiogenesis mainly through the regulation of hypoxia-inducible factor 1α (HIF-1α)-mediated or NF-κB-mediated expression of vascular endothelial growth factor (VEGF) in cancer tissues [12,142]. Briefly, Pin1 promotes the stability and transcriptional activity of HIF-1α in human cancers or diseases [143,144,145]. Pin1 reverses the inhibitory mechanism of PML-mediated suppression of HIF-1α-induced angiogenesis in clear cell renal cell carcinoma and prostate cancer [106,146]. Pin1 also induces angiogenesis through stabilization of HIF-2α in colorectal cancer cells [147]. Pin1 upregulates NF-κB expression and consequently promotes angiogenesis through increased VEGF expression in a number of cancers [80,81,83]. Furthermore, the other VEGF promoting transcriptional factors, such as β-catenin and FoxM1, are upregulated by Pin1 to drive angiogenesis in various cancers [148,149,150,151].

### 3.6. Pin1 Promotes Invasion and Metastasis

Cancer invasion and metastasis are critical cancer capacities for a primary tumor progressing to higher malignant grades. The best characterization of a cancer cell in the beginning is a loss of E-cadherin expression, which lets tumor cells have an anti-quiescence ability and a higher motility [152,153]. Loss of E-cadherin drives epithelial-mesenchymal transition (EMT), a developmental regulatory program, which promotes local invasion and distal metastasis [154,155,156,157]. Pin1 expression is higher in metastatic cancer compared with primary cancer [158]. Increased observations have indicated that Pin1 plays a critical role in the promotion of invasion and metastasis in many cancers [21,159,160].

An increasing body of evidence shows that β-catenin not only increases cell proliferation but also promotes migration and invasion in cancer cells [66,157,161]. Therefore, Pin1 promotes invasion, metastasis and even other malignant tumor processes partly through the upregulation of β-catenin-mediated signaling cascades [19,68,150]. Transforming growth factor (TGF)-β plays a critical role in tumor metastasis [162,163,164,165] and Pin1 has been found to upregulate TGF-β by stabilizing its mRNA [166]. Furthermore, Pin1 directly promotes TGF-β-induced migration and invasion in human prostate cancer cells [167].

Growing evidence has demonstrated that NF-κB plays an important role in the induction of EMT and the promotion of tumor metastasis [168,169,170]. Pin1 could promote metastasis via activation of the NF-κB-interleukin (IL)-18 feedback loop in pancreatic cancer cells [171]. Pin1 also promotes EMT through upregulation of STAT3 and NF-κB in human gallbladder cancer [172]. Additionally, NOTCH signaling drives metastasis in numerous cancers [173,174,175,176] and Pin1 prevents NOTCH1 and NOTCH4 from undergoing FBXW7-mediated degradation, leading to an increase in breast cancer stem cells’ (CSCs) self-renewal and metastasis [177]. In addition to protein stability, Pin1 also activates NOTCH1 transcriptional activity by facilitating γ-secretase-mediated cleavage and release of the active intracellular domain. Interestingly, NOTCH1 signaling upregulates Pin1 expression, thereby generating a positive feedback loop for malignant progression in human breast cancers [31]. Pin1 has also been reported to enhance EMT and chemoresistance by upregulating FoxM1 and the Wnt/β-catenin signaling pathway in cervical cancer cells [130]. Additionally, Pin1 promotes invasion and metastasis through activation of p53M and BRD4 in many cancers [88,178].

In addition to EMT, cytoskeleton reorganization affects cell motility including invasion and metastasis [179,180,181,182]. Protein tyrosine phosphatase (PTP)-PEST, along with FAK and Src, cooperate to regulate the phosphorylation and dephosphorylation of focal adhesion proteins, which control focal adhesion turnover and stimulate cell motility in normal cells and cancer cells [183,184]. Furthermore, Pin1-mediated isomerization of PTP-PEST facilitates the interaction between PTP-PEST and FAK and the dephosphorylation at Y397 on FAK, which in turn promotes cell migration, invasion and metastasis [185,186]. HDAC6-mediated deacetylation of α-tubulin regulates microtubule-dependent cell motility [187,188,189]. Pin1 upregulates HDAC6 expression through stabilization of HDAC6 mRNA and protein, which consequently promotes cell motility in cancer cells [190,191]. Moreover, overexpression of Pin1 promotes centrosome amplification [24,192], which increased centrosomal microtubule nucleation and Rac1 activity, which disrupts normal cell–cell adhesion and promotes invasion in cancer cells [193,194]. It also implies that major tumor capabilities proceed these cross-interactions.

### 3.7. Pin1 Drives the Genome Instability and Mutations of Cancer

The goal of cell division for most cells is to accurately duplicate the genome and then equally segregate the duplicated genome into two daughter cells having the same genetic material as their parent cell. Genomic instability is defined as an increase tendency of alterations in the genome. During the life-course of a single cell, the genome is constantly damaged by myriad exogenous and endogenous stimuli. To maintain genome stability, DNA damage response (DDR) is adopted to sense and repair DNA damage in cells. Genome instability acts a major driving force to tumorigenesis. Therefore, most cancer is tolerant to a certain level of genome instability and mutations. In cancer cells, Pin1 not only represses the DNA damage-induced senescence and apoptosis but also promotes the genome instability such as aneuploidy and centrosome amplification.

Damage-induced DNA double-strand breaks (DSBs) can be repaired using error-free homologous recombination (HR) and error-prone nonhomologous end-joining (NHEJ) [195]. CtIP interacts with the MRE11 complex, and consequently promotes DNA end resection to proceed homologous recombination [196]. However, Pin1 promotes CtIP degradation and facilitates error-prone NHEJ leading to increase genome instability [197]. Pin1 is regarded as a regulator to regulate DSB repair through CDK-mediated modulation of DNA-end resection [198]. Meanwhile, CDK-induced centrosome amplification drives genome instability [199]. Additionally, rapid proliferation leads to increased replication stress activates DNA damage and generation of structural and numerical chromosome instability (CIN) partially through centrosome amplification in cancer cells [200,201].

In addition to accurate replication, the equal segregation of the replicated genome into daughter cells is also crucial for genome stability. Therefore, the aberrant segregation and division processes would contribute to genome instability. Separase controls the equal segregation of sister chromatid by hydrolyzing cohesion [202]. Pin1-mediated isomerization modulates separase proteolytic activity during mitosis [203]. The centrosome protein 55 kDa (Cep55) plays a key role in cytokinesis. Furthermore, cytokinesis failure usually results in tetra-ploidy leading to genomic instability. Pin1 could regulates cytokinesis through Cep55 [204]. Pin1 also affects the SEPT9-mediated separation of daughter cells [205].

### 3.8. Pin1 Facilitates Tumor-Promoting Inflammation

The immune cells, largely of the innate immune system in the tumor microenvironment, have been known to have important tumor-promoting effects on neoplastic progression by providing bioactive molecules into the tumor microenvironment, including growth factors, survival factors, proangiogenic factors, extracellular matrix-modifying enzymes, and inductive signals that lead to driving EMT and other cancer-promoting programs [61,206,207,208].

Pin1 is a pivotal regulator for inflammation. The dysregulation of Pin1-mediated inflammation contributes to many diseases, including cancer. Pin1 promotes IL-18-mediated tumor-promoting inflammation and pancreatic cancer progression in pancreatic ductal adenocarcinoma (PDAC) via upregulation of NF-κB signaling [171]. Interleukin receptor-associated kinase M (IRAK-M) induced by TGF-β, an inactive kinase, is predominantly expressed in tumor-associated macrophages and acts a potent negative regulator of Toll-like receptors (TLRs) signaling, thereby promoting tumor growth [209]. Furthermore, Pin1 also promotes the nuclear translocation of IRAK-M and induces selected proinflammatory genes expression to enhance the IL-33-induced airway inflammation in dendritic cells [210]. IL-22 is a modulator for proliferation and inflammation via activation of ERK, JNK and STAT3. Pin1 is found to regulate IL-22-induced MAP3K8-mediated activation of ERK, JNK and STAT3 for promoting cancer-associated inflammation in the tumor microenvironment [211]. NADPH oxidase-mediated superoxide production plays a key role in host defense and inflammation in human neutrophils. Pin1 promotes TLR7/8 agonist CL097-mediated priming of fMLF-induced NADPH oxidase cytosolic component p47phox phosphorylation, leading to superoxide production [212].

Besides, granulocyte-macrophage colony-stimulating factor (GM-CSF) is a crucial hematopoietic growth factor and immune modulator involved in activation of various circulating leukocytes [213]. Eosinophil-mediated GM-CSF production drives short and long-term fibrotic pathology in asthma. Furthermore, Pin1 regulates the AU-rich element-binding proteins AUF1 and hnRNP C with GM-CSF mRNA to control the stability of GM-CSF mRNA, respectively, in eosinophils and T lymphocytes [214,215]. In addition to elevating tumor-promoting, Pin1 also upregulates some anti-inflammatory factors, such as NUR77 and glucocorticoid receptor (GCR) to regulate inflammation response [216,217].

### 3.9. Pin1 Governs the Metabolic Reprogramming of Cancer

The characteristics of neoplastic diseases involve not only uncontrolled cell proliferation but also distinct adjustments of cellular energetics to support cell growth and division. Normal cells rely primarily on glycolysis in cytosol and oxidative phosphorylation in mitochondrial to generate the energy for cellular processes under aerobic conditions; while under anaerobic conditions, anaerobic glycolysis is favored to generate pyruvate, thereby converting to lactate. In contrast to normal cells, most neoplastic or highly proliferative cells instead rely on aerobic glycolysis even in the presence of oxygen. The reprogramming of glucose metabolism is a phenomenon termed the “Warburg effect” [218]. Cancer cells direct the metabolic switch to promote survival, growth, proliferation, and long-term maintenance through rapid ATP synthesis, increased biosynthesis, established tumor associated microenvironment, enhanced ROS-induced signaling transduction and accelerated genome instability [219].

Mounting evidence suggested that Pin1 plays a crucial role in metabolic reprogramming via controlling the function of critical factors involved in aerobic glycolysis in cancer cells [220]. Pin1 enhances the stability of β-catenin and activates Wnt/β-catenin signaling to regulate tumor metabolic reprogramming [221]. Pin1 also promotes the stability and transcriptional activity of HIF-1α to act a central regulator of glycolysis, cancer metabolism and cancer cell proliferation [222]. Interestingly, aerobic glycolysis also prevents the aerobic degradation of HIF-1α protein, activates HIF-1α transcriptional activity, and enhances the expression of several HIF-1-activated genes to promote tumorigenesis [223]. Additionally, Pin1 promotes the stability and activity of Myc, thereby regulating cell growth and metabolism in cancers [224]. Pin1-mediated mitochondria translocation of PGK1 activates pyruvate dehydrogenase kinase 1 (PDHK1) to inhibit the pyruvate dehydrogenase (PDH) complex, leading to enhanced glycolysis in the cytosol accompanied by reduced mitochondrial pyruvate metabolism [225]. Pyruvate Kinase M2 (PKM2) is a critical mediator of aerobic glycolysis in cancer cells. Pin1 promotes PKM2 binding to importin α5 and nuclear translocation, which cooperates with β-catenin to induce c-Myc expression and modulate metabolism [226]. Therefore, targeting the metabolic reprogramming is a feasible strategy for cancer therapy [227,228].

### 3.10. Pin1 Advances Cancer Cells to Evade Immune Destruction

As for tumor formation, immune surveillance is a monitoring process of the immune system to recognize and eradicate all initial cancer cells and nascent tumors. Therefore, the immune system contributes to the surveillance against tumors [229]. However, many patients with an apparently normal immune system still develop cancers. This indicates that tumor cells appear to somehow have biocapacities to avoid attack by the immune system or are able to evade the extent of immunological killing, thereby escaping eradication. Therefore, three essential phases for the interaction between host and tumor cells have been proposed: elimination, equilibrium and escape [230]. In addition to resisting or eradicating incipient neoplasias, the immune system has been proven to promote tumor progression and malignancy [231]. Mounting evidence shows that Pin1 plays a crucial role in the regulation of immune response [12,232].

Interferon-gamma (IFN-γ)-induced indoleamine 2,3-dioxygenase (IDO) expression protects the fetus from maternal immunity. Similarly, IDO is found prevalently overexpressed in tumors and suppresses T cell immunity, leading to tumor growth in the tumor microenvironment [233]. Ligation of CTLA-4 by CD80/CD86 activates the PI3K/AKT and NOTCH pathway to stimulate IDO production in dendritic cells [234]. In the meanwhile, NOTCH-induced Pin1 expression promotes casein kinase II-mediated PTEN suppression, thereby increased PI3K/AKT signaling and IDO production [234]. Additionally, Pin1 upregulates TGF-β through stabilizing its mRNA [166], which acts a crucial regulator of immune responses, leading to immune escape for cancer development [235].

Interleukin-1 receptor activated kinases (IRAKs) are key mediators in the signaling pathways of TLRs/IL-1 receptors (IL-1Rs) in regulating the innate immune system [236]. Upon activation of TLR7 and TLR9, Pin1 triggers IRAK1 activation and release from the receptor complex, leading to IRF7 activation and type I interferons production [18]. In addition, IFN regulatory factor 3 (IRF3) is an important transcription factor involved in the regulation of innate immune responses [237]. IRF3 stimulates IFN-β expression for antiviral immune response [238]. However, Pin1 suppresses the transcriptional activity and promotes ubiquitin-mediated downregulation of IRF3 leading to decreased IFN-β production [239]. Therefore, Pin1 can modulate immune surveillance from multiple pathways.

## 4. Pin1 Targeted Therapies for Cancer Treatment

### 4.1. Targeting Pin1 Could Suppress Tumor Growth

Cancer is the leading cause of human death worldwide [240] and the rate of cancer occurrence as well as mortality are still growing globally. In the past, scientists tried to treat cancer by developing inhibitors, which were highly targeted to a single pathway. However, multiple dysregulated pathways are concomitantly involved in tumorigenesis [61]. Therefore, a new generation of anti-cancer drugs which are able to inhibit multiple cancer-driving pathways or a combination of therapies could be a more suitable strategy for cancer treatment [189]. Pin1 is highly expressed in numerous cancers, including breast, lung, hepatic, prostate, colorectal, glioblastoma and esophageal cancers. Pin1 participates in the diverse cancer biocapacities mentioned above. Therefore, it is an attractive drug target.

Mounting evidence indicates that inhibition of Pin1 is an effective means to reduce cell proliferation and repress tumorigenesis. As we mentioned previously, a cell can control Pin1 expression through transcriptional, post-transcriptional and post-translational regulation which in turn regulates the physiological or pathological processes. Tumor suppressors, such as p53, Rb and BRCA1, can directly suppress Pin1 expression to reduce its tumor-driving processes. Several miRNAs including miR-140-5p, miR-200b, miR-200c, miR296-5p, miR-370, miR-628-5p and miR874-3p inhibit tumor progression by facilitating the degradation of Pin1 mRNA. However, Pin1 reduces miRNAs biogenesis by modulating the function of XPO5 in cancers, leading to downregulation of total miRNA expression profiling in tumors. In addition to regulating the expression of Pin1, Aurora kinase A, and protein kinase A could block the substrate binding ability of Pin1 by phosphorylating its Ser16 residue in the WW domain. Furthermore, DAPK1 inhibits Pin1 by phosphorylating Ser71 in the PPIase domain, thereby suppressing malignant processes. Due to Pin1 acting as a crucial hub-mediated cancer-driving signaling pathway, Pin1 inhibitors have been discovered and synthesized for cancer treatment. Most drugs identified target the catalytic PPIase domain, as well as a few targeting the WW domain. These potential Pin1 inhibitors are listed in Table 1.

Juglone is an allelopathic compound identified firstly as Pin1 inhibitor. The Juglone modifies the sulfhydryl group of cysteines to inhibit Pin1 activity and suppress cell proliferation in various cancer cells including breast cancer, lung cancer, hepatocellular carcinoma, and prostate cancer [241,242,243,244,245]. Moreover, a high dose of juglone treatment even reduces Pin1 protein expression [246]. Juglone also regulates the functions of other proteins by inhibiting RNA polymerase II and preventing PP2A-mediated dephosphorylation of Rab4 for mitotic progression [41,247]. However, juglone harbors diverse specificities, which limits its potential application for cancer treatment.

PiB, a fused tetracyclic tetra-one that was screened from a chemically synthesized library, is able to inhibit Pin1 (IC50 = 1.5 μM) [248]. Unlike juglone, PiB is a competitive inhibitor of Pin1 that blocks the proliferation of any Pin1-expressing cells, but not that of Pin1-deficient cells. Additionally, PiB treatment reduces Nonog expression through transcriptional and post-translational level mRNA, consequently suppressing the self-renew ability and teratomas-forming ability of the cancer stem cells [249]. TME-001, a dual inhibition of Pin1 (IC_50_ = 6.1 μM) and cyclophilin (IC_50_ = 13.7 μM), was discovered from a high-throughput screening of chemical libraries and its administration suppresses the cell proliferation of HeLa cells [250].

All-trans retinoic acid (ATRA) was discovered from a mechanism-based high-throughput screening system, which inhibits the Pin1 activity by covalently binding to the catalytic domain of Pin1 [251]. ATRA treatment not only induces the degradation of Pin1 protein, but also contributes to suppressing oncogenic function by downregulation of cyclin D1 in breast cancer cells. ATRA administration also triggers the degradation of PML-RARα oncoprotein, leading to suppression of proliferation in acute promyelocytic leukemia (APL) cells and mouse models. In this context, ATRA is regarded as a targeted therapy for APL and breast cancer. Currently, the Food and Drug Administration (FDA) approved ATRA for acute promyelocytic leukemia (APL) therapy. Moreover, ATRA possesses potent anticancer activity against hepatocellular carcinoma (HCC) by blocking multiple cancer-driving pathways to reduce tumorigenicity in a mice model of HCC [252].

Arsenic trioxide (ATO) is a compound associated with transcriptional regression of Pin1. At present, Arsenic trioxide as medicament is approved for APL therapy by the FDA. Specifically, the combine therapy of ATRA with ATO can effectively treat APL, since ATRA administration promotes uptake of ATO through upregulation of aquaglyceroporin 9, a transmembrane protein involved in arsenic uptake [253]. It is also characterized by ATO inhibition and degradatioin of Pin1, thereby suppressing the oncogenic pathways by noncovalent binding to Pin1’s active site [254]. Furthermore, co-treatment of ATO and ATRA, at clinically safe doses, synergistically blocks numerous Pin1-mediated oncogenic pathways and provides an attractive strategy to fight APL, breast cancer or other diseases.

The inhibitive effect of KPT-6566 on the catalytic activity of Pin1 was identified by a mechanism-based screening [160]. KPT-6566 is an organic compound containing a polycyclic aromatic hydrocarbon that is able to covalently bind to the sulfanyl-acetic acid group and tertbutyl-phenyl group through a sulfonamide moiety. KPT-6566 covalently docks in the catalytic pocket of Pin1 to inactivate its PPIase activity. Similar to ATRA and ATO, KPT-6566 inhibits Pin1 signaling and targets it for degradation. Unlike other Pin1 inhibitors, KPT-6566 not only possesses anti-proliferation activity, but also holds cytotoxic effects on cancer cells through the generation of reactive oxygen species and DNA damage. Therefore, KPT-6566 treatment triggers apoptosis and suppresses cell proliferation in a variety of cancer cells [160]. Moreover, KPT-6566 has also been demonstrated to specifically execute an anti-proliferative effect on Pin1-expressing cells but not on Pin1-silenced cells. KPT-6566 treatment has been found to reduce lung metastasis in a mouse model of breast cancer.

6,7,4′-trihydroxyisoflavone (6,7,4′-THIF), a major metabolite of daidzein, inhibits esophageal cancer growth. Pin1 was one of the candidates of the 6,7,4′-THIF targeted proteins. The 6,7,4′-THIF interacts with the WW domain and PPIase domain of Pin1 and blocks its emzyme activity [255]. Pyrimidine derivatives were identified to inhibit Pin1 activity by covalent binding to Pin1 [256]. (S)-2 treatment reduces cyclin D1 expression and evokes cytotoxicity in human prostate cancer PC-3 cells through inhibiting Pin1 by covalent binding with Cys113 of Pin1 in the catalytic domain [257].

Pin1 inhibitors are urgently needed for treating cancer diseases. Equally, advancing in drug delivery and target to Pin1 is crucial for effective cancer treatments. Although some compounds display a more potent effect against Pin1 PPIase activity in vitro, their insolubility in water is a hurdle limiting their application for clinical use. Moreover, further pre-clinical and clinical studies are also needed to evaluate the safety and efficacy of Pin1 inhibitors for clinical cancer therapy.

**Table 1:** Peptidyl-prolyl cis-trans isomerase NIMA-interacting 1 (Pin1) inhibitors.

Pin1 Inhibitor	Chemical Structure	Inhibitory Mechanism and Significance	References
Juglone; 5-Hydroxy-1,4-naphthalenedione	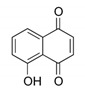	Covalently bond to PPIase catalytic domain	[246,258]
PiB; 1,3,6,8-tetrahydro-1,3,6,8-tetraoxo-benzo[lmn][3,8]phenanthroline-2,7-diacetic acid, 2,7-diethyl ester	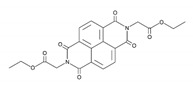	Competitive inhibitor of Pin1 PPIase catalytic domain	[248]
TME-001; 2-(3-chloro-4-fluoro-phenyl)-isothiazol-3-one	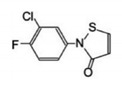	Dual inhibitor of Pin1 and cyclophilin. Competitive inhibitor of Pin1 PPIase catalytic domain	[250]
All-trans retinoic acid (ATRA); (2E,4E,6E,8E)-3,7-dimethyl-9-(2,6,6-trimethylcyclohexen-1-yl)nona-2,4,6,8-tetraenoic acid	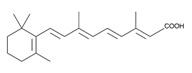	Competitive inhibitor of Pin1 PPIase catalytic domain The FDA approved ATO in combination with the ATRA for first-line treatment of low-risk acute promyelocytic leukemia	[251]
Arsenic trioxide (ATO); arsenic(3+);oxygen(2-)	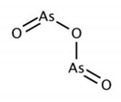	Competitive inhibitor of Pin1 PPIase catalytic domain The FDA approved ATO in combination with the ATRA for first-line treatment of low-risk acute promyelocytic leukemia	[254]
API-1; N-[[4-[(2-amino-7H-purin-6-yl)oxymethyl]phenyl]methyl]-2,2,2-trifluoroacetamide	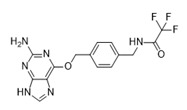	Competitive inhibitor of Pin1 PPIase catalytic domain	[259]
KPT-6566; 2-[4-(4-tert-butylphenyl)sulfonylimino-1-oxonaphthalen-2-yl]sulfanylacetic acid	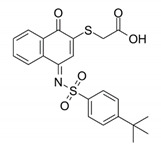	Covalently bond to Pin1 PPIase catalytic domain	[160]
6,7,4′-THIF; 6,7-dihydroxy-3-(4-hydroxyphenyl)chromen-4-one	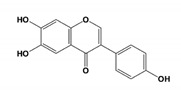	Directly interacts with Pin1 at the WW and PPIase domains to reduced PPIase activity and substrates binding	[255]
Pyrimidine derivatives	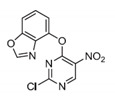	Covalently bond to Pin1 PPIase catalytic domain	[256]
(S)-2	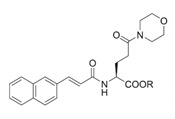	Covalently bond to Pin1 PPIase catalytic domain	[257]
EGCG; [(2R,3R)-5,7-dihydroxy-2-(3,4,5-trihydroxyphenyl)-3,4-dihydro-2H-chromen-3-yl] 3,4,5-trihydroxybenzoate	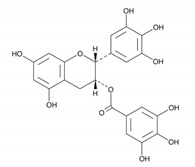	Bind both WW and PPIase domains	[260]
Cyclic peptide	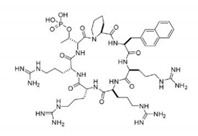	Substrate analogs for Pin1	[261]
Cis-locked alkenepeptidomimetics	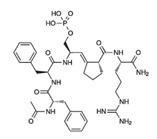	Substrate analogs for Pin1	[262]
PEPTIDE	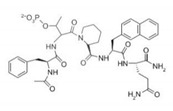	Competitive inhibitor of Pin1 PPIase catalytic domain	[263]
Benzo-thiophene	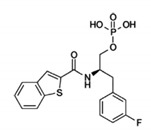	Competitive inhibitor of Pin1 PPIase catalytic domain	[264]
Phenyl-imidazoes	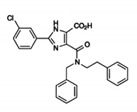	Competitive inhibitor of Pin1 PPIase catalytic domain	[265,266]
AKBA derivative	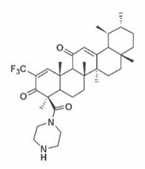	Bind to WW domains	[267]
TAB29; 3-phenyl-4,6-bis(phenyl-methoxy)-1-benzofuran	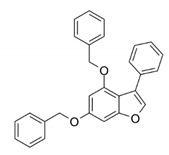	Competitive inhibitor of Pin1 PPIase catalytic domain	[268]
Benzimidazolederivative	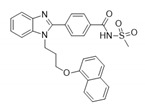	Bind to Pin1 PPIase catalytic domain	[269]

### 4.2. Targeting Pin1 Is Effective to Prevent Tumor Recurrence

Drug resistance and tumor metastases are the two leading causes of cancer-related morbidity and mortality [270,271]. Cancer cells develop resistance to targeted drugs by drug inactivation, altered drug metabolism, change in drug transport and targets, evolving DNA damage repair, cell death inhibition, the epithelial-mesenchymal transition (EMT), epigenetic alterations and tumor cell heterogeneity [272]. These mechanisms can work independently or in combination to contribute drug resistance. Intriguingly, Pin1 acts as a crucial hub to drive the ten major cancer-promoting biocapacities mentioned above. These parameters are closely related.

Glowing evidence indicates that Pin1 can advance the ten major biocapacities to bypass or combat the drug-mediated signaling pathways. (1) In respect to drug inactivation, many anticancer drugs must undergo metabolic activation in order to acquire clinical efficacy. Profoundly, Pin1 is used to catalytically modify or facilitate the degradation of the associated proteins needed to process the pro-drug. Actually, Pin1 could also disrupt the complex formation of the drug leading to drug inactivation. BRCA1/2 is prevalently mutated in breast cancer patients. Synthetic lethality from a combination with PARP inhibitor and BRCA1/2 mutation has been adopted to treat breast cancer. However, BRCA1-proficient cells are resistant to PARP inhibitor treatment due to Pin1-mediated BRCA1 maintaining genome stability upon DNA damage. Luo et al. found that Pin1 inhibition sensitizes BRCA1-proficient breast cancer in PARP inhibition treatment [273]. (2) As to alteration of the drug target, a drug’s efficacy is dependent on the status of its molecular targets and effectors such as mutations or modifications of expression levels which lead to drug resistance. Tubulin polymerization inhibitors, including taxanes, taxol and vinca alkaloids, disrupt the microtubule arrays and cause cell cycle arrest. Subsequently, cell cycle arrest at G2/M activates CDK1, which results in phosphorylation of LATS (large tumor suppressor) and conforms to a Pin1 binding motif. Interaction of Pin1 and LATS results in activating YAP and TAZ-mediated signaling against tubulin, depolymerizing drug-induced apoptosis. Conversely, inhibition of Pin1 sensitizes cancer cells to tubulin depolymerizing drugs [274]. (3) In regard to drug efflux, upregulation of organic anion transporter facilitates cancer cells dumping out the drug. In contrast, depletion of Pin1 sensitizes MDA-MB-231 cells to paclitaxel partially through downregulation of multidrug resistance (MDR) genes (MDR1 and MRP4) [275]. (4) To improve DNA damage repair, many chemotherapy drugs directly or indirectly damage DNA to induce cell death. Silencing of DNA damage repair is an important action enhancing the pharmacological action of anticancer drugs. Pin1-mediated BRCA1 can maintain genome stability and promote DNA repair responding to anticancer-drug-caused DNA breakage. Therefore, Pin1 has an important role in the evolution of drug resistance partially through a decline in DNA repair efficiency and fidelity [273]. (5) As to cell death inhibition, drug-induced cancer cell death is one of the fundamental strategies of cancer chemotherapy. Pin1 promotes drug resistance leading to the tumor cell evading anticancer stimulated cell death. The tumor cell gaining drug-resistance to anticancer drugs represents not only the inhibition of intrinsic and extrinsic pathways for acquiring apoptosis-resistance, but also the activation of cell survival signaling, as mentioned in Section 3.1. (6) As to epithelial-mesenchymal transition (EMT), it is a regulatory mechanism by which solid tumors render metastasis. EMT promotes changes in the cancer cell and in the stromal cells that form the local environment. EMT also initiates angiogenesis, which is responsible for forming new blood vessels around metastatic tumors that supply myriad nutrients and oxygen, as well as evacuating metabolic wastes and carbon dioxide for survival. Mounting evidence shows that Pin1 promotes drug resistance through enhancing EMT [276,277,278]. (7) For tumor cell heterogeneity, genome instability is one of the cancer hallmarks which is a critical factor. A fraction of the cells with heterogeneity in the cancer cell population have stem cell-like properties and are usually drug resistant. Under a certain stress, drug resistant cancer cells survive and can expand, which contribute to pathology over time. Pin1 is known to promote centrosome amplification which contributes to genome instability. In addition, Pin1 promotes stemness-driving signaling. Reversely, inhibition of Pin1 reduces the ratio of cancer stem-like cells and increases drug sensitivity of cancer cells [277]. In summary, upregulation of Pin1 promotes drug resistance and survival in cancer cells.

**Figure 3:**
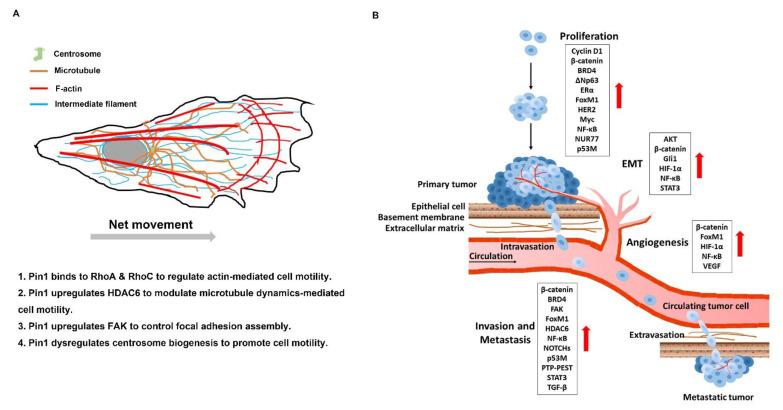
Pin1 is involved in cell motility and tumor metastasis. (**A**) Cell morphology and motility are tightly regulated by cytoskeleton components. Pin1 promotes cell motility via multiple pathways, such as the enhancement of focal adhesion dynamics, actin- or microtubule-mediated cell migration, and centrosome amplification-mediated Rho GTPase activity and increased microtubule nucleation. (**B**) In addition to the activation of proliferative signaling cascades, Pin1 induces angiogenesis to help establish the primary tumor. Pin1 further promotes the intravasation of cancer cells from the primary tumor into the circulatory system through upregulation of epithelial-mesenchymal transition (EMT)-related genes and invasion-associated genes. Pin1-driving cell motility-associated genes upregulation facilitates the colonization of circulating tumor cells and the formation of metastatic tumor in distal regions or organs.

Cell motility is mechanism directing and moving towards chemoattractant gradients (directional) for cell growth and survival [279]. For tumor cells, cell motility pathologically contributes to tumor cell survival and growth, as well as metastatic spreading. Pin1 drives EMT progress which is significantly involved in metastatic ability [154]. However, Pin1 plays a pivotal role in control of cell motility not only through the EMT program but also through global regulation of cytoskeleton reorganization (Figure 3A). It is known that Pin1 can interact with RhoA and RhoC and activates their activity, thereby enhancing actin-dependent cell motility [23,133]. In addition to actin, microtubule dynamics is a critical way to control cell motility. HDAC6 controls the acetylation of α-tubulin to regulate microtubule dynamics [187]. Evidence shows that Pin1 promotes microtubule-mediated cell motility through upregulation of HDAC6 [190,191]. Furthermore, upregulation of Pin1-induced centrosome overduplication promotes Rac1-mediated cell invasion [193]. Pin1 also promotes PTP-PEST dephosphorylate FAK to control the focal adhesion turnover and stimulate cell migration, invasion, and metastasis [185,186]. Therefore, inhibition of Pin1 is an effective strategy to modulate cell motility in cancer.

Metastasis is composed of serial processes [280]. Briefly, the spreading activity of tumor cells relies upon cell motility, which leads to local invasion into neighboring connective tissue, then entry into nearby blood or the lymphatic vessels (intravasation), exit of tumor cells from the vessels (extravasation), and migration of tumor cells into distant tissues and expansion of the metastatic colonies into macroscopic tumors (colonization) [61,281]. A growing number of works have demonstrated the invasion- and metastasis-promoting functions of Pin1 in human cancer. The multiple roles of Pin1 in metastatic processes are illustrated in Figure 3B. Pin1 promotes proliferation of clots into the small nodules of cancer cells and gradually develops a primary tumor through sustaining proliferation signaling. Then Pin1 upregulates the angiogenesis-associated factors to increase VEGF expression which triggers neovascularization to support rapid expansion of cancer cells. Pin1 promotes EMT progression and disruption of basement membrane, and consequently enhances intravasation of cancer cells into the circulation system. Pin1 enhances the expression of the invasion and metastasis-associated factors leading to increased migration of circulating tumor cells (CTCs). It also promotes invasion of CTCs into the distal tissue. The disseminating cancer cells proliferate and colonize in the distal organs through sustaining proliferation signaling and finally form a metastatic tumor [282]. Therefore, inhibition of Pin1 is an effective strategy to prevent metastases.

## 5. Conclusions

Cancer is a complex disease with many dysregulated biological pathways and, simultaneously, multiple genetic and epigenetic alterations. Targeted therapies with high efficacy and low side effects are pharmacological criteria to treat cancers. However, tumor relapse is a clinical challenge. Problematically, tumor recurrence significantly attributes to drug resistance and metastases. According to the hallmarks of cancer summarized by Hanahan and Weinberg, we understand the multiple biocapacities for tumorigenesis. Therefore, they all are therapeutic targets for cancer treatment.

Pin1 overexpression is a pathological feature in numerous cancer tissues and CSCs, and is correlated with poor prognosis in various cancer patients. Cancer cells harboring Pin1 overexpression have phenotypic centrosome amplification, leading to genome instability and higher heterogeneity. Intriguingly, Pin1 almost acts as the center to regulate the multiple cancer-driving processes in Pro-directed phosphorylation dependent pathways. Furthermore, Pin1 also upregulates multiple pathways responsible for drug resistances. Therefore, it is a potential crucial therapeutic target. Although there are many identified Pin1-targeted inhibitors, which exert anticancer activity in in vitro experiments, in animal models and even in some APL patients, many Pin1 inhibitors share poor aqueous solubility which limits their clinical applications. Besides, a delivery system improving the bioavailability of Pin1 inhibitors is also urgently needed for development in animal models. More effort should be made to fill these gaps. In another way, Pin1 inhibitors might be an adjuvant not only to promote the efficacy of combined targeted therapies but also to prevent drug resistance. Advancing the pharmacological strategy for Pin1 inhibition, the pre-clinical and clinical studies are also needed to evaluate the safety and efficacy of Pin1 inhibitors for cancer therapy.

## Data Availability

Not applicable.
